# Characterizing Antimicrobial Resistance in Clinically Relevant Bacteria Isolated at the Human/Animal/Environment Interface Using Whole-Genome Sequencing in Austria

**DOI:** 10.3390/ijms231911276

**Published:** 2022-09-24

**Authors:** Adriana Cabal, Gerhard Rab, Beatriz Daza-Prieto, Anna Stöger, Nadine Peischl, Ali Chakeri, Solveig Sølverød Mo, Harald Bock, Klemens Fuchs, Jasmin Sucher, Krista Rathammer, Petra Hasenberger, Silke Stadtbauer, Manuela Caniça, Peter Strauß, Franz Allerberger, Markus Wögerbauer, Werner Ruppitsch

**Affiliations:** 1 Austrian Agency for Health and Food Safety, 1096 Vienna, Austria; 2 Institute of Hydraulic Engineering and Water Resources Management, Technical University of Vienna, 1040 Vienna, Austria; 3 Institute for Land and Water Management Research, Federal Agency for Water Management, 3252 Petzenkirchen, Austria; 4 Institute of Chemical, Environmental and Bioscience Engineering, 1060 Vienna, Austria; 5 Center for Public Health, Medical University Vienna, 1090 Vienna, Austria; 6 Section for Food Safety and Animal Health Research, Department of Animal Health, Welfare and Food Safety, Norwegian Veterinary Institute, 1433 Ås, Norway; 7 National Reference Laboratory of Antibiotic Resistances and Healthcare Associated Infections, Department of Infectious Diseases, National Institute of Health Doutor Ricardo Jorge, 1600-609 Lisbon, Portugal; 8 Department of Biotechnology, University of Natural Resources and Life Sciences, 1180 Vienna, Austria

**Keywords:** antimicrobial resistance, One Health, whole genome sequencing, antimicrobial resistance genes

## Abstract

Antimicrobial resistance (AMR) is a public health issue attributed to the misuse of antibiotics in human and veterinary medicine. Since AMR surveillance requires a One Health approach, we sampled nine interconnected compartments at a hydrological open-air lab (HOAL) in Austria to obtain six bacterial species included in the WHO priority list of antibiotic-resistant bacteria (ARB). Whole genome sequencing-based typing included core genome multilocus sequence typing (cgMLST). Genetic and phenotypic characterization of AMR was performed for all isolates. Eighty-nine clinically-relevant bacteria were obtained from eight compartments including 49 *E. coli*, 27 *E. faecalis*, 7 *K. pneumoniae* and 6 *E. faecium*. Clusters of isolates from the same species obtained in different sample collection dates were detected. Of the isolates, 29.2% were resistant to at least one antimicrobial. *E. coli* and *E. faecalis* isolates from different compartments had acquired antimicrobial resistance genes (ARGs) associated with veterinary drugs such as aminoglycosides and tetracyclines, some of which were carried in conjugative and mobilizable plasmids. Three multidrug resistant (MDR) *E. coli* isolates were found in samples from field drainage and wastewater. Early detection of ARGs and ARB in natural and farm-related environments can identify hotspots of AMR and help prevent its emergence and dissemination along the food/feed chain.

## 1. Introduction

Antimicrobial resistance (AMR) in bacteria is a leading cause of death worldwide, with *Escherichia coli*, *Staphylococcus aureus* and Klebsiella pneumoniae being the top three resistant pathogens [1]. In the European region alone, the incidence of bacterial AMR in 2019 was equal to 67.7 per 100,000 people and according to new estimations, the global number of deaths associated with AMR may reach up to 10 million by 2050 [2,3]. For Austria in particular, the AURES resistance report 2020 showed that the resistance rates in human *E. coli* and *K. pneumoniae* isolates remained stable (high) in comparison to 2019, or decreased for fluoroquinolones, third generation cephalosporins and aminoglycosides. The rate of *K. pneumoniae* resistance to carbapenemases, for example, remained stable in 2020 compared to the previous year in contrast to the rest of Europe. For Enterococci, the resistance rate to aminopenicillin and aminoglycosides remained stable or decreased, respectively, while MRSA continued its decreasing trend over the last 5 years [4].

In 2017 the World Health Organization (WHO) published a list of antibiotic-resistant “priority pathogens“ including three categories ranked from 1 to 3 [5]. These categories referred to the greater or lesser need to develop new antibiotics to treat infections caused by the bacteria on this list, whose treatment options are increasingly limited by the emerging resistance [6]. Priority 1, named *critical* priority or known by the acronym ESKAPE, grouped Gram-negative carbapenem-resistant and/or extended-spectrum beta-lactamases (ESBL)-producing *Enterobacteriaceae*. Priority 2, also named *high* priority, included diverse Gram-negative fluoroquinolone-resistant bacteria, such as *Salmonellae* and Gram-positive vancomycin-resistant bacteria, such as *Enterococcus faecium* or methicillin-resistant *S. aureus*. Lastly, priority 3 bacteria also included both Gram-negative and positive bacteria of medium priority, such as penicillin-resistant *Streptococcus pneumoniae.* In order to combat AMR, surveillance studies on clinically-relevant bacteria included in the abovementioned WHO list are increasingly incorporating One Health approaches [7]. One Health is a multidisciplinary concept created in 2004 that aims to sustainably balance and optimize the health of people, animals and ecosystems [8]. Several drivers of geographical, ecological, anthropogenic and agricultural nature modulate the intra and interrelationships of the One Health components [9].Thus, this holistic approach is essential for reducing the emergence and spread of resistance and maintaining the effectiveness of antibiotics. Antimicrobial resistance genes (ARGs) are naturally present in the environment, but antibiotic usage selects for antibiotic resistant bacteria (ARB) and, hence, for ARGs [6]. However, less attention is paid to the environment, with most studies focusing exclusively on human and the animal (mostly livestock) components. Evidence shows that some ARGs such as the *bla*_CTX_ encoding for extended spectrum betalactamases have their origin in bacterial species that are common in the environment and these can easily be transferred to human and animal bacteria [10]. Likewise, wildlife is to some extent ignored in the surveillance of clinically-relevant bacteria [11].

To our knowledge, no studies have been performed in Austria to monitor the occurrence of ARB in the three One Health components simultaneously using whole genome sequencing (WGS). To address this issue, we aimed in this study to assess the phenotypic and genetic resistance of six bacterial species included in the WHO priority list of antibiotic-resistant bacteria across environmental compartments representing the food/feed chain.

## 2. Results

### 2.1. Isolates by Species and Compartment

Out of 61 samples collected from nine compartments over a one-year-growing period, a total of 89 clinically-relevant isolates were obtained from 40 of those samples belonging to eight compartments and were selected for phenotypic and genotypic AMR analysis. In the other 19 samples, only non-clinically relevant isolates were obtained and were therefore excluded from the study. Forty-nine (55.1%) of the clinically-relevant isolates were *E. coli*, 27 (30.33%) were *E. faecalis*, 7 (7.86%) *K. pneumoniae* and 6 (6.74%) *E. faecium*. No MRSA nor *Salmonella* spp. isolates were found. By compartment, wildlife (*n* = 29) and wastewater (*n* = 28) accounted for the majority of the isolates (Table 1). One *E. coli* isolate was obtained from feed and no clinically-relevant species were detected in crops. Wastewater had the highest species diversity, comprising four of the abovementioned bacterial species, followed by groundwater, with three species. *E. coli* was found in all compartments except crops. *E. faecalis* was found in all compartments except crops, feed and field drainage water. *E. faecium* was isolated from ground- and wastewater and *K. pneumoniae* only from wastewater.

Most of the isolates had been retrieved from samples collected in July (n = 30), September or October (14 and 18 respective isolates), with E. coli being more than half (n = 16) of those (Figure S1).

### 2.2. Phylogenetic Relationships

**Figure 1:**
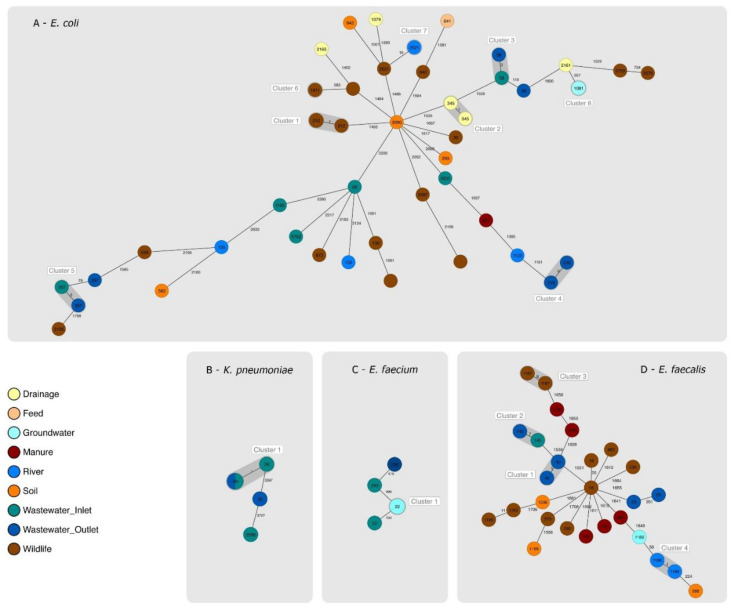
Genetic relatedness between isolates of the same species based on their core genomes. STs are indicated inside each circle, which represents one or more isolates. Empty circles correspond to isolates with new STs. Clusters are highlighted in grey. Colors identify the compartment from which each isolate was gained. The number of allelic differences between two or more isolates is shown on the connecting lines between them. (**A**) Minimum spanning tree (MST) displaying the collected *E. coli* isolates; (**B**) MST displaying the collected *K. pneumoniae* isolates; (**C**) MST displaying the collected *E. faecium* isolates; (**D**) MST displaying the collected *E. faecalis* isolates.

The 49 *E. coli* isolates resolved into 36 STs and 44 cgMLST (CT) types. Three isolates presented new STs, and all isolates belonged to a novel CT (Table S1). We detected four cgMLST clusters (clusters 1, 3, 4 and 5) and four pseudo-clusters (clusters 2, 6, 7 and 8) (Figure 1A).

Cluster 1, grouped four *E. coli* ST212 wildlife fecal isolates collected in September from two different fecal samples. Cluster 3 grouped two ST58 isolates from two different wastewater subcompartments (inlet and outlet), which had been collected in July. Cluster 4 grouped two wastewater ST216 isolates from two different wastewater samples (subcompartment outlet) collected in July and October. Cluster 5 grouped two ST357 wastewater isolates collected in January 2021 from the inlet and the outlet. Among the singletons, we detected major STs such as ST69 and ST1193, both from wastewater.

The seven *K. pneumoniae* isolates were resolved into three STs (20, 29 and 3586). Five ST29 isolates obtained from five different samples and three sampling dates (April, July and October 2020) were grouped in a cluster. Three had been obtained from the inlet and two from the outlet (Figure 1B and Table S2).

The six *E. faecium* isolates were resolved into three STs (22, 236 and 293). One cgMLST pseudo-cluster with three ST22 groundwater isolates was detected. Another ST22 isolate, not clustering with any other isolate, was obtained from an inlet wastewater sample (Figure 1C and Table S3).

For *E. faecalis*, the 27 isolates were resolved into 11 known STs and eight new STs. One cgMLST two-isolate cluster was detected (cluster 2), which had two ST145 wastewater isolates. One isolate had been obtained from a wastewater sample from the outlet and another one from the inlet. Clusters 1, 3 and 4 were pseudoclusters (Figure 1D and Table S4).

### 2.3. Phenotypic AMR

The screening for antimicrobial susceptibility by disk diffusion detected 29 (32.6%) resistant isolates to at least one of the antimicrobials tested. One *E. coli* isolate (id: 510423-20) was identified as an ESBL producer (amoxicillin-clavulanic acid diameter >5 mm with respect to that of the cefotaxime).

By E-test, the overall prevalence of AMR was 29.2% (26/89), including the resistance to those antimicrobials tested after confirming ARG presence by WGS. In total, 13.4% (*n* = 12) of the isolates were resistant to one antimicrobial, 10.1% (*n* = 9) to two antimicrobials, 2.24% (*n* = 2) to three antimicrobials, 2.24% (*n* = 2) to six antimicrobials and one isolate (1.12%) was resistant to 10 antimicrobials. There were 63 isolates (70.8%) susceptible to all antimicrobials tested. E-test patterns of phenotypic resistance by species are given in Table 2.

Overall, only macrolide- and tetracycline-resistant *E. coli* and *E. faecalis* isolates were detected in soil, feed and manure samples from farmers 1 and 2.

In *E. coli*, all the cluster-related isolates were confirmed as susceptible to all tested antimicrobials. Among singletons and pseudo-clusters, resistance to tetracycline (*n* = 6, MIC: 32–128 µg/mL), ampicillin (*n* = 5, MIC: ≥256 µg/mL), streptomycin (*n* = 5, MIC: 32–192 µg/mL), ciprofloxacin (*n* = 3, MIC: 8–32µg/mL), moxifloxacin (*n* = 3, MIC: 8 µg/mL), trimethoprim-sulfamethoxazole (*n* = 3, MIC: ≥32 µg/mL), amoxicillin-clavulanate (*n* = 1, MIC: 24 µg/mL), azithromycin (*n* = 1, MIC: ≥256 µg/mL), cefotaxime (*n* = 1, MIC: ≥32 µg/mL), erythromycin (*n* = 1, MIC: ≥256 µg/mL) and gentamicin (*n* = 1, MIC: 32 µg/mL) were found. One isolate showed intermediate resistance to cefepime (*n* = 1, MIC: 4 µg/mL).

All *K. pneumoniae* (*n* = 7) presented low-level ampicillin resistance (MIC: 12–24 µg/mL) and the five ST29-cluster isolates were highly resistant (HLAR) to fosfomycin (MIC: ≥1024 µg/mL). All isolates were susceptible against amikacin, cefoxitin, colistin, ertapenem and kanamycin.

Nine *E. faecalis* isolates out of 27 were at least resistant to tetracycline (MIC: 8–96 µg/mL). Two of those were also resistant to streptomycin (MIC: 768 µg/mL) and kanamycin (MIC: ≥256 µg/mL), one to erythromycin (MIC: ≥256 µg/mL) and another one to clindamycin (MIC: 24µg/mL).

Among *E. faecium* we only detected one resistant isolate, which presented low-level resistance to ampicillin (MIC: 64 µg/mL).

### 2.4. ARGs, VGs and Plasmids

Species-specific differences were observed in ARG content between the isolates. Thus, *E. coli* isolates carried a mean of 46.3 ARGs, followed by *K. pneumoniae*, with 20.6 ARGs, *E. faecium* with 3.3 and *E. faecalis,* with 2.4 each. By compartment, isolates obtained from drainage samples carried, on average, 48.6 genes, followed by feed (47 genes), river (33 genes), wildlife (32.1 genes), soil (28 genes), wastewater (25.8 genes), groundwater (14.1 genes) and manure (11.8 genes).

After categorizing the ARGs by drug class, the largest group in all four species was the *multidrug* group, accounting for 70% of the ARGs in *E. faecium*, 65.28% in *K. pneumoniae*, 54.7% in *E.coli* and 34.38% in *E. faecalis*. The second most frequently found drug class varied among species. Thus, aminoglycosides represented 30% of the ARGs found in *E. faecium*, diaminopyrimidines represented 32.9% of the ARGs in *E. faecalis*, followed by tetracyclines (18.8%), while betalactams grouped 18.1% of the ARGs in *K. pneumoniae*. All other drug classes were represented by less than 10% of ARGs in all four species.

**Figure 2:**
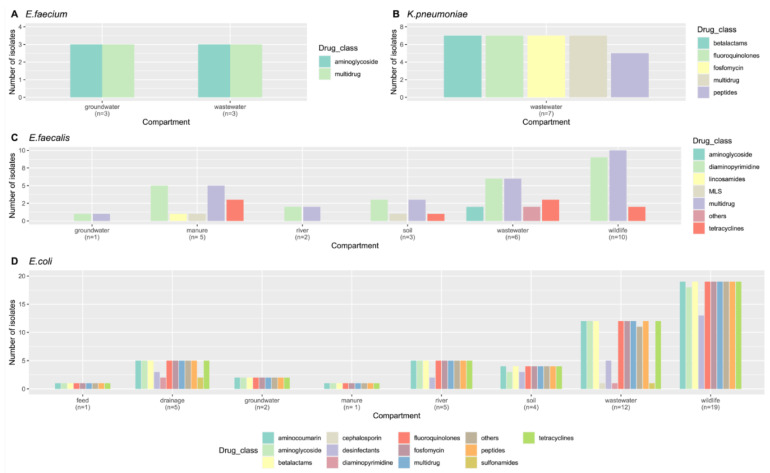
Number of isolates obtained in each species ((**A**)—*E. faecium*, (**B**)—*K. pneumoniae*, (**C**)—*E. faecalis*, (**D**)—*E. coli*) and compartment, harboring ARGs allocated to different drug classes.

Overall, manure and wastewater isolates were the most diverse, with ARGs in five or more different drug classes (Figure 2). The proportion of isolates presenting ARGs in each of the above-described categories also varied among species but was similar between compartments and collection dates.

All ARGs, VGs and plasmids detected in each isolate by species and compartment are shown in Tables S1–S4. The presence and absence of a given ARG in each isolate can also be visualized in Figure S2a–d.

In *E. coli* isolates, ARGs associated with intrinsic resistance such as the *AcrA* efflux system, chromosomal *ampC* or *amp*C-like genes or target replacement genes such as *bacA*or *eptA*, among others, were detected in all isolates. Acquired aminoglycoside ARGs were detected in six (12.2%) isolates, five of which were confirmed as streptomycin-resistant. Two of those isolates were ST345 obtained from drainage water (ids: 510655-20, 510656-20) and carried *aadA2* and *aph(6)-Id* (also known as *strB*) genes, while one isolate of ST1193 (id: 510423-20) belonged to a wastewater-inlet sample and carried *aac(3)-IId, aadA5, aph(3”)-Ib* and *aph(6)-Id*. This isolate had been also confirmed as resistant to gentamicin. Two other isolates from soil (meadow; id: 510520-20) and manure (id: 510473-20), which were confirmed as moderately resistant to streptomycin, carried *aph(3”)-Ib* and *aph(6)-Id* and *aph(3”)-Ib* alone. Lastly, one streptomycin-susceptible isolate from wastewater (id: 510667-20) carried *aph(3”)-Ib* and *aph(6)-Id*. The six tetracycline-resistant isolates carried *tet* genes, including *tetA* or *tetB* plus *tetR*, and five of them (ids: 510655-20, 510656-20, 510520-20, 510473-20, 510667-20) were carrying aminoglycoside genes. The sixth tetracycline-resistant isolate was obtained from a feed sample (id: 510063-21). In addition, the abovementioned drainage isolates (ids: 510655-20, 510656-20) and wastewater isolate (id: 510423-20) carried either *dfrA12* or *dfrA17* genes and the three harbored the sulfonamide genes *sul1* and *sul2*, being resistant to trimethoprim-sulfamethoxazol. In addition, these three isolates had point mutations in *gyrA* (D87N) and *parC* (S80I) associated with resistance to moxifloxacin and ciprofloxacin and carried the betalactamase gene *bla*_TEM-1_ responsible for ampicillin resistance. *Bla*_TEM-1_ was also found in a wastewater-inlet isolate (id: 510245-20), which was also phenotypically resistant to ampicillin. The wastewater isolate 510423-20 harbored a *bla*_CTX-M-55_ as well, which had a transposase *IS26* followed by an *ISECp1* 287bp upstream from the start of the gene. Downstream from the betalactamase gene, a WubC-encoding gene was detected. This was the cefepime and cefotaxime resistant isolate, which also carried the macrolide resistant gene *mph*A linked to its high level resistance to azithromycin. Lastly, one fosfomycin susceptible soil isolate from a field fertilized with pig manure (id: 510299-20) carried the fosfomycin gene *fosA2*.

Overall, three *E. coli* isolates were designated as MDR (id: 510423-20, 510655-20, 510656-20), since they carried acquired ARGs from five to seven drug classes and were confirmed as phenotypically resistant to all of them (id: 510423-20, 510655-20, 510656-20). The MDR isolate from wastewater (id: 510423-20) carried up to four plasmids with ARGs, namely an IncB/O/K/Z conjugative plasmid carrying *aac(3)-IId*, *aadA5, bla*_CTX-M-55_*, mph(A)*, *sul1* and *dfrA17*, a col(pHAD28) mobilizable plasmid carrying *aph(3’’)-Ib, aph(6)-Id* and *sul2*. For the two drainage isolates (ids: 510655-20, 510656-20) the presence of a mobilizable plasmid incQ1 harboring *aadA2, aph(3”)-Ib, aph(6)-Id, bla*_TEM-1B_*, sul1, sul2, tet(A)* and *dfrA12* genes was confirmed. The manure isolate 510473-21 carried a conjugative plasmid with *aph(3”)-Ib, aph(6)-Id* and *tet(A),* while the soil isolate 510520-21 carried *aph(3”)-Ib* and *aph(6)-Id* in a conjugative plasmid.

In *K. pneumoniae*, where low-level resistance to ampicillin was found in all isolates, chromosomally-encoded *bla*_SHV_ (*bla*_SHV-187_, *bla*_SHV-11_) and *amp*C-like (*ampH*) genes were detected. The fosfomycin genes *fosA5* and *fosA6* were found in two and five isolates. Only those carrying *fosA6* were resistant. We did not find evidence of *fosA*-carrying plasmids.

In *E. faecium*, the only phenotypically-resistant isolate (510426-20 ampicillin resistant) harbored up to thirteen simultaneous known point mutations and four unknown mutations in the chromosomal gene *pbp5*. None of the isolates carried acquired ARGs. All (*n* = 6) carried the chromosomal *msr*C, associated with macrolide–streptogramin B resistance, *aac(6′)-Ii*, responsible for low-level resistance to aminoglycosides and *efm*A, which codes for an efflux pump associated with macrolide and fluoroquinolone resistance. No known mutations in the 23S ribosomal genes associated with linezolid resistance were found. Likewise, neither linezolid (*cfr, optrA, poxtA*) nor vancomycin ARGs (*van* clusters) were detected.

In *E. faecalis*, the most frequently detected ARGs were the lincosamide gene *lsa*, which was present in all isolates, the dihydrofolate reductase gene *dfrE* (21/27) and *efrA* encoding for an ABC multidrug efflux pump (22/27). In addition, acquired tetracycline genes were detected in all the nine tetracycline-resistant isolates (*tetM*: 9/27 and *tet45*: 3/27). Two of those isolates (ids: 510672-20, 510673-20) were the ones confirmed as resistant to streptomycin and kanamycin by E-test and carried the aminoglycoside genes *aad(6)* and *aph(3”)-IIIa* and the streptothricin resistance gene *sat-4,* which were encoded on a mobilizable plasmid. The *tet* genes of those isolates were also integrated in mobilizable plasmids. Additionally, the erythromycin resistance gene *ermB* was detected in two unrelated isolates from manure (id: 510691-20) and soil (id: 510295-20). In both isolates the *ermB* was located in the transposon *Tn917*. The soil isolate was the only one confirmed as phenotypically resistant to erythromycin. The manure isolate carried the *ermB* together with tetracycline genes on a conjugative plasmid, while the soil isolate carried those genes on a mobilizable plasmid. The lincosamide resistance gene *lnuG* was detected in an isolate obtained from pig manure (id: 510683-20) located in the transposon *tn6260*. The isolate presented a MIC for clindamycin of 24 µg/mL.

In *E. coli*, the most frequently detected phylogroup was B1 (*n* = 28, 57.7%), followed by B2 (n = 8, 16.3%), D (*n* = 6, 12.2%), A (*n* = 5, 10.2%) and E (*n* = 2, 4.1%). Isolates of different phylogroups were found in different compartments (Table S3). *E. coli* phylogroup B2 isolates had, on average, 54 VGs, E isolates 38 VGs, and D, B1 and A isolates had 19, 13 and 9 respective VGs. Of the isolates, 90% had fimbrial-related genes. Thirteen isolates from the phylogroups B1, B2 and D were considered as potentially pathogenic. Seven out of the eight B2 isolates identified carried the *chuA, fyuA* and *vat* genes and were classified as UPEC. One of them was the MDR isolate 510423-20. The remaining B2 isolate, obtained from a meadow sample (soil), together with two B1 wildlife isolates were classified as atypical EPEC (ids: 510307-20, 510398-20, 510620-20), since they carried *eae* gene but not *bfpA*. In addition, one of the B1 isolates from wildlife was identified as a new STEC (id: 510632-20), which carried the *stx2c* variant, the enterohaemolysin gene *ehxA*, the subtilase gene *subAB*, was *eae*-negative and belonged to serotype O142:H16. The ST was newly designated as ST12789. Lastly, two isolates from wastewater (id: 510245-20) and wildlife (id: 510402-20-WH) were identified as presumptive ExPEC, which carried the ExPEC-specific markers *kpsMII* and *iutA*. The isolate from wastewater belonged to the major *E. coli* group ST69 and was one of the ampicillin resistant isolates. In total, 36 isolates were designated as commensal and did not carry the required marker combinations to be considered as *E. coli* pathotypes.

In *K. pneumoniae*, VGs were detected in all the seven isolates, including enterobactin (*n* = 7, 100%), salmochelin (*n* = 7, 100%) and yersiniabactin genes (*n* = 1, 14.3%). The aerobactin gene *iuc* and the hypermucoid phenotype gene *rmpA* were not detected in any isolate. Additionally, isolates had capsule types typical of non-hpkv strains (capsular types other than K1 and K2).

In *E. faecium*, the only VG detected was the adhesin-encoding gene *acm*.

Regarding *E. faecalis*, the presence of VGs varied among the isolates. *BopD, cpsA, cpsB, ebpA, ebpB, ebpC, efaA* and *srtC* genes were detected in all the 27 strains. Other frequently found genes (>70%) were EF0818, *fss1*, *gelE* and *sprE*. *Cyl* genes were found in only two isolates from river water (ids: 510416, 510417) and two isolates from wastewater (ids: 510672, 510673). The latter also had the *asa1* gene. The *esp* gene coding for a surface protein was not found in the isolates.

## 3. Discussion

In this study, we aimed to obtain clinically-relevant bacteria from connected environmental compartments. We detected conjugative and mobilizable plasmids and other mobile genetic elements that can spread via horizontal gene transfer. Although we only found resistances to macrolides and tetracyclines in isolates obtained from samples directly associated with farmers 1 and 2 (manure, soil and feed), we found resistances and ARGs in other connected compartments such as the field drainage, mainly associated with veterinary drugs. This highlights the importance of monitoring for AMR and ARB emergence on farms and farm-related environments.

Overall, our work has used a One Health approach, allowing us to detect four of the six targeted clinically-relevant bacterial species, some of which carried acquired ARGs, were phenotypically resistant to one or more antimicrobials and/or were present in several samples. Although there is recent evidence that pig manure increases the persistence of Salmonella sp. in the soil, from where it can penetrate into crops, we did not find *Salmonella* spp. in any of the compartments studied. Nevertheless, certain types of soil and plants seem to be more associated with persistence and more frequent detection of *Salmonella* [12]. Likewise, previous reports have confirmed that up to 11% of the MRSA acquired in the community come from crops fertilized with pig manure [13]. However, we did not detect MRSA in our HOAL samples. A reason for this could be that the prevalence of MRSA is lower in small-scale farm systems compared to industrial ones [14].

We obtained a higher number of isolates in July. This observation does not seem to be related to the application of pig manure to the field, which occurred in March, and could be due to the diverse representation of compartments among the samples collected in July.

In addition, only *E. faecalis* and *E. coli* were detected in the pig manure, but these were also the two most abundant species. Therefore, it is difficult to assess the role of pig manure on the bacterial population with so many potential factors affecting the diversity, and the same applies to the ARGs detected in the isolates. A recent longitudinal study also performed at the Austrian HOAL observed that the concentrations of a set of ARGs detected by qPCR decrease after manure application reaching the baseline levels in the sampled soils quickly [15]. Here, we did not observe differences between isolation dates and the diversity of ARGs in the isolates, but differences between isolates of different species and compartments, independent of the point in time. Thus, a study on other non-clinically relevant species would be of interest in order to assess the ARGs that are naturally present (not anthropogenic origin) in environmental bacteria, which can be hotspots of AMR with or without increased selective pressure from human-derived activities.

The low prevalence or absence of clinically-relevant bacteria in feed and crops detected here is in accordance with previous reports [16]. This and the geographical location of the HOAL, far away from big complex urban areas and the size of pig farms, might explain these results.

In general, isolates from manure and wastewater were more often phenotypically resistant to the antimicrobials tested than the isolates gained from other compartments, and had more ARGs per isolate. In addition, we observed that for these two compartments, ARGs detected could be distributed in a wider range of drug class categories than those from other compartments. This indicates that manure and wastewater isolates, and in particular, *E. coli*, presented more ARGs, both intrinsic and acquired, contributing to a higher degree of phenotypic AMR. *E. coli* isolates also showed high diversity in terms of STs, with three isolates having unknown STs. The detection of four clusters composed of isolates obtained from samples of different compartments and/or isolation dates among such a diverse isolate collection and low sample size confirms the spread of different clones between compartments, some of which could be pathogenic. ST357 (cluster 5), classified as possible UPEC here, has been found to be one of the most frequent UPEC clones causing UTI in humans [17]. Cluster 3, composed by ST58 wastewater isolates, has been reported in different hosts and sources, including wastewater, as one of the top 20 ExPEC clones able to cause urosepsis [18] and it is newly described as a possibly zoonotic ST [19]. However, we reported it here as commensal, since it did not fulfill the criteria for ExPEC designation. According to the literature, isolates that carry ≥2 or more of *chuA, fyuA, vat,* and *yfcV* genes are considered as UPEC, while isolates that carry ≥2 or more of *apaH*/*papC*, *sfa-focDE*, *afa-draBC*, *iutA* and *kpsMII* are considered as ExPEC [20]. ST212 (cluster 1-wildlife) is not only a major *E. coli* clone but has been associated with wildlife, in particular vultures, and has been reported to disseminate across the human–animal-environment interface [21]. In addition, it has been detected in patients with UTI, although here we described it as commensal [22]. In agreement with other authors, ST216 was described here as commensal *E. coli*, matching their phylogroup (B1) and source of isolation with those in the literature, where they were found in the environment and wildlife [23,24]. Among the singletons, the detection of an UPECST1193 O75:H11 MDR clone was remarkable. ST1193 is an emerging fluoroquinolone resistance strain genetically related to the MDR pandemic clone ST131 [25,26], not detected in our study. The sequence of our ST1193 isolate was described in our previous report [26] and differed by only 13 alleles from an UPEC isolated from a male patient with urinary tract infection in Australia in 2011 (Uberstrain accession: ESC_PA1198AA, strain MS10745) and by 17 alleles from a MDR strain isolated in the US causing neonatal meningitis (accession: CP030111). Although the Australian strain was also MDR and carried most of the acquired ARGs detected in our strain (*sul1/sul2,* *bla*_TEM-1_*, aph(6)-Id, aac(3)-IId, qacE*), it lacked *bla*_CTX-M-55_, therefore being cephalosporin susceptible. In addition, we detected a ST69 isolate from wastewater, which is also a major ExPEC clone that can cause sepsis and urinary tract infections [18]. In agreement with other authors, ST69 carried *bla*_TEM-1_ and was susceptible to extended-spectrum cephalosporins [27]. However, we did not find the *mcr-9* gene, which has been associated with this clone in particular [28]. We also found an *eae*-negative STEC O142:H16 isolate (id: 510632-20). Recently, an STEC strain belonging to the same serotype but of an unknown source was sequenced by CDC (SRR14150468), differing from our strain by 65 alleles in the core genome. While our strain was isolated from a wildlife fecal sample from a brown hare (*Lepus europaeus*), there are other reports of this serotype obtained from raw milk, mozzarella cheese [29] and free ranging deer [30]. Like the deer isolate, our isolate lacked *eae* and had the *stx2c* variant, *ehxA* and other virulence genes such as *subAB,* indicating that it was potentially pathogenic for humans [31]. In addition, the three isolates that had *eae* genes but no shigatoxin can be considered in the absence of *stx* genes and *bfpA* as aEPEC [32], but could be equally pathogenic, since some aEPEC such as the O103, also detected here in a soil sample, have been linked to human disease but originated from animals [33].

As for AMR in our *E.coli* isolates, it can be seen in general that those strains with acquired ARGs mostly expressed a resistant phenotype associated with the ARG in question. An exception for this was the only AMC resistant isolate (id: 510298-20), which was also AMP resistant, and for which no *bla_TEM_* gene was found. AMC and AMP resistance are known to be associated with TEM-1 overproduction, which suggests that in the absence of this gene, hyperproduction of the chromosomal AmpC may occur, causing the AMC-AMP resistant phenotype [34]. It is also noteworthy that one of the farmers interviewed had administered tetracyclines, sulfonamides and penicillins, coinciding with the resistances seen in most of the resistant *E. coli*. Indeed, tetracycline ARGs but also aminoglycoside genes, a drug class not administered to the HOAL pigs in principle, were detected in isolates from at least three compartments simultaneously. According to the European medicines agency antimicrobial expert group (EMA-AMEG), the use of aminoglycosides in animals should be limited to scenarios when no other alternative antimicrobials can be used, since the development of resistances to this drug class represents a high risk to public health [35]. Different types of enzymes, such as those encoded in the *aadA2* or *aad5* genes, confer a lower level of resistance to streptomycin, while those encoded in the *aph(3”)-Ib* and *aph(6)-Id* confer a higher level of resistance. Accordingly, we detected combinations of the two aminoglycoside genes that corresponded with the highest resistance, while strains with only one type of gene were more susceptible to this antibiotic. Moreover, the gentamicin-resistant isolate (id: 510423-20) was the only one carrying *aac(3”)-IId*, a determinant for gentamicin resistance, while the absence of resistance markers such as *aph(3’)-Ia*, which is responsible for kanamycin resistance, explained the phenotypic susceptibility against this antimicrobial [36,37]. Isolates carrying *tet* genes were obtained from five compartments, including pig manure, and highlight that the use of tetracyclines in pigs select for these tetracycline genes [38]. Moreover, in wildlife isolates the only ARGs detected were *tet* genes. This lack of AMR in wildlife contrasted with a previous study carried out in Germany, where although low, AMR against cephalosporins, fluoroquinolones and colistin was detected [39].

*K. pneumoniae* was only detected in wastewater samples, although this species is adapted to other environments such as soil and vegetation [40]. Despite the low number of retrieved isolates, we were able to identify a cluster of five ST29 isolates from five different samples and three sampling dates, which indicates the persistence of this clone over time and in both the inlet and the outlet of the wastewater treatment plant. Unlike in previous studies, our ST29 isolates did not carry ESBL genes, which seems to be uncommon in this ST [41]. In any case, the discharge of *K. pneumoniae* clones (MDR and/or pathogenic) via the effluent of the wastewater treatment plant into a downstream body of water and further on to the crops via irrigation using river water cannot be excluded.

Regarding phenotypic resistance, the low-level ampicillin resistance observed in all the *K. pneumoniae* isolates is known to be of an intrinsic origin and encoded in the chromosome by *bla*SHV-1 and *ampH* genes [42,43], both detected in our isolates. The high level of fosfomycin resistance observed in five of our isolates was most likely associated with *fosA6* genes encoded in the chromosome. Research shows that these genes can be located either in the chromosome or in plasmids via mobilization through *IS10* in both *K. pneumoniae* and *E. coli*, and there is prior evidence of high level fosfomycin resistance due to *fosA6* in *E. coli* [44,45]. However, *IS10* was not found in any of these isolates in the surroundings of the *fosA6* gene nor was a plasmid detected, pointing to a chromosomal location. On the contrary, the two *fosA5* isolates were fosfomycin-susceptible, in agreement with other authors, and although IncR and/or one IncFIB_K plasmids were detected in these isolates, they did not harbor the *fos*A5, therefore indicating a possible chromosomal location [46].

Although all *K. pneumoniae* isolates carried enterobactin genes and the *iroE* gene, which is part of the locus that encodes for salmochelin, in order to designate isolates as hvKp, at least four of the following genes need to be present in a *K. pneumoniae* isolate: *rmpA, rmpA2*, *iucA*, *iroB* and *peg-344* [47,48]. Moreover, other authors differentiated commensal *K. pneumoniae* from hvKp due to the presence in hvKp of salmochelin and aerobactin [49], as well as the presence of specific capsule serotypes for hvKp [50]. None of the isolates detected here matched the hvKp definition, in accordance with the VGs found.

All the ARGs found in *E. faecium* isolates are known to be intrinsic resistance determinants in this species [51,52], which is in agreement with the lack of phenotypic resistance found here, except for one ST236 isolate, whose ampicillin resistance was due to point mutations in *pbp5* genes. In contrast, other authors obtained non-resistant isolates of this ST from environmental samples [53]. Conversely, other works have reported ST22 isolates obtained from human and animal sources as *optrA* carriers [54], while our ST22 isolates were not phenotypically resistant nor did they carry any ARG. Regarding the only VG found in *E. faecium*, the *acm* gene, this has recently been described in clinical strains of patients with endocarditis, indicating pathogenic potential.

In this study, we found a high diversity of STs for *E. faecalis*, some of which have been described as zoonotic. Indeed, ST16 and ST40 isolates identified here in wildlife and wastewater have been detected before in hospitalized and non-hospitalized patients, food production animals, wild birds and the environment, namely wastewater [51,55,56,57].

Regarding AMR, *E. faecalis* is known to be intrinsically resistant to lincosamides via efflux pumps, all cephalosporins, quinupristin-dalfopristin, low level aminoglycosides and trimethoprim [52,58]. Here, all *E. faecalis* isolates carried *lsa*, responsible for intrinsic resistance to lincosamides and streptogramin A [59]. However, the detection of *lnu(G)* in a pig manure isolate could also explain the clindamycin MIC of 24 µg/mL observed, which is ten times higher than previously reported [60]. *Lnu(G)* was first identified in swine located on the lincosamide nucleotidyl transferase transposon *Tn6260* of *E. faecalis.* Unlike lincomycin, which is frequently used in veterinary medicine, clindamycin is often prescribed in human medicine. Therefore, the detection of *lnu(G)* in pig manure could lead to an increase in the resistance to lincosamides when disseminated and acquired via the food/feed chain. The *ermB* gene, detected in another manure and a soil isolate could be responsible for the erythromycin resistance detected in the manure isolate. Interestingly, the pig manure from which this isolate was gained belonged to the same farmer, as did the crop field from which the soil sample was taken (corn fertilized with manure). As reported elsewhere [61], pig manure can be the origin of *ermB* and *lnu(G)* in *E. faecalis* obtained from soil at nearby pig farms.

In agreement with recent reports, most of our *E. faecalis* isolates carried *dfrE*, which, unlike *dfrF,* is considered intrinsic [62,63]. Conversely, only the two *E. faecalis* isolates from wastewater showing high level streptomycin and kanamycin resistance had acquired aminoglycosides genes, namely, *aph(3’)-IIIa* [64]. As for tetracycline resistance, the *tetM* gene, a resistance marker that can be transferred by HGT [65] was detected here and it has been proved to increase in soils fertilized with manure. Indeed, in addition to three manure isolates carrying *tetM* in our study, the only tetracycline resistant *E. faecalis* obtained from soil belonged to a field fertilized with manure.

Regarding VGs in *E. faecalis*, isolates with more VGs (>23) were from wildlife feces or pig manure, while in *E. coli*, wastewater isolates carried more VGs (>45). Among the *E. faecalis* VGs typically associated with disease in humans, we only detected three of them, namely *asa1, cylA* and *gelE*, alone or in combination with each other [66]. In agreement with other authors, *gelE* was the most frequent VG among that group of genes, while *cylA* was not detected in animals, but in the environment (river and wastewater) [66]. Nevertheless, isolates with more VGs(>23) did not carry *cylA* nor *asa1*, and at the same time, these and other VGs have also been isolated from *E. faecalis* obtained from healthy humans. Thus, caution is advised when designating isolates as pathogenic [67].

In conclusion, we have demonstrated that some clones are detected at different time-points, but we have not seen the same clone in more than one compartment. We detected the same ARGs in distinguishable isolates from different compartments, most of them as phenotypically resistant to at least one antimicrobial. In addition, although the HOAL symbolizes the exchange of ARGs and ARBs between different compartments existing in nature, the mere presence of two pig farms in this HOAL is not representative of the totality of existing farming practices. Different results might be obtained when choosing industrial breeding farms, which would contribute more significantly to the environmental antibiotic resistance pool. Additionally, the presence of larger urban centers near the HOAL would have a greater impact on ARG exchange. Likewise, it is uncertain whether some ARGs have been exchanged between isolates of different compartments after applying manure to the field or if this is purely coincidental. Taken together, the detection of the same ARGs in different compartments warrants further attention. Moreover, some of the gaps in our knowledge can be better addressed by using culture-free methodologies such as shotgun metagenomics in addition to WGS on the bacterial cultures, in order to characterize the environmental resistome, as well as the microbial diversity in the different compartments.

## 4. Materials and Methods

### 4.1. Sampling

**Figure 3:**
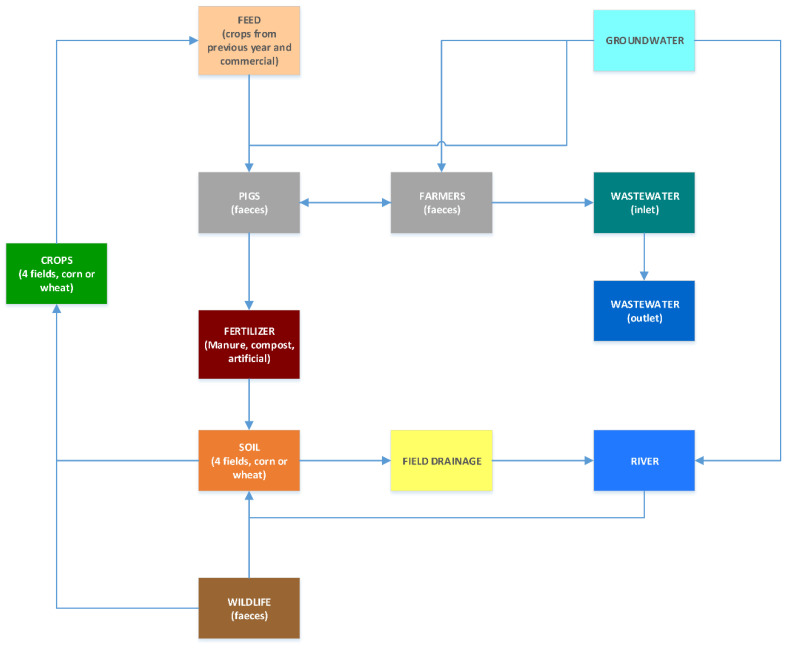
Graphical representation of the relationship between the different HOAL compartments from which samples were collected. Grey compartments are those from which no sample could be obtained.

As part of the OHEJP FED-AMR project, we collected 61 samples from nine out of 11 different but interconnected environmental compartments in an annual longitudinal study (Figure 3) from March 2020 to January 2021 in the area of Petzenkirchen, Austria.

**Figure 4:**
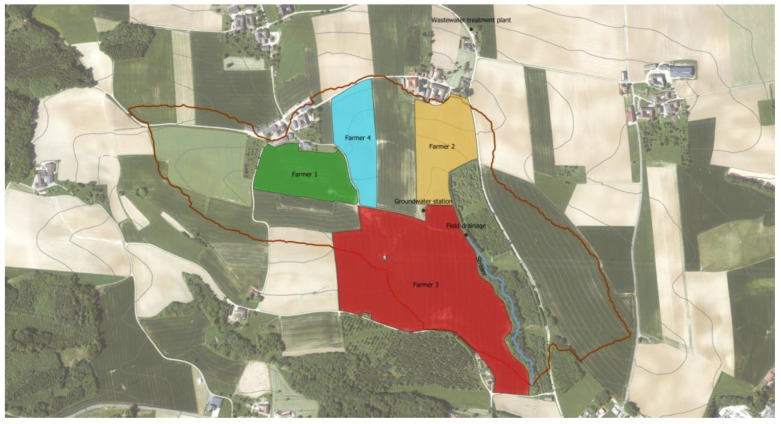
Aerial view of the HOAL in Petzenkirchen, Lower Austria. The limit of the HOAL is depicted by the red line. The sampled fields are colored by farmer. Green, yellow and red fields are sown with corn. The field in blue is sown with wheat. The blue line represents a stream (i.e., compartment “river”). Alongside there is a field drainage and groundwater station. The wastewater treatment plant is outside the catchment area, next to the house of farmer 2.

The sampling area was part of a hydrological open-air laboratory (HOAL) (Figure 4). The intended purpose is to understand water flow related processes by investigating their microorganisms [68].

Three corn fields and one winter wheat field, each from a different farmer, were chosen for collecting soil and crop samples. Two of the corn fields were fertilized with pig manure plus artificial fertilizer (farmer 1 and 2), while the third one was nourished with compost plus artificial fertilizer (farmer 3). The winter wheat field (farmer 4) was enriched only with artificial fertilizer. In the catchment area, there were two pig farms from two different farmers (1 and 2). Data for the use of antibiotics on these two farms were retrieved from the official veterinary records (Table S5). Feed given to the pigs originated from a mixture of crops from the previous year (2019). These crops had received artificial fertilizer, manure or combination of both. Additionally, farmers purchased commercial feed with additives (mineral feed). At the time of the first sampling, pig manure was less than a year old and the slurry pits were emptied every autumn. That is, pig manure was a mixture of manure accumulating since fall of the previous year. Fertilization either with swine manure, compost or commercial artificial fertilizer took place in March–April 2020.

Next to farmer 3’s field was a field drainage and a small river from which samples were also collected. Wildlife feces were easily accessible from the ground of the field nourished with compost (farmer 3), the largest field. In addition, samples were taken from the wastewater treatment plant collecting the effluents of nine agricultural households right outside the catchment area; two of these belonged to farmers 1 and 2. Samples from ground water supplying these households and the pig farms for both human and animal consumption were also obtained. The sample collection procedure for each compartment and the transportation conditions are available at Zenodo (doi:10.5281/zenodo.5081756).

### 4.2. Isolate Selection and Sequencing

From the samples, 0.5 g were introduced in 15 mL sterile tubes with glass beads and 4.5 mL 0.1% buffer Na_4_P_2_O_7_.10H_2_O buffer at pH 7.0. Serial dilutions 1:10 were made and 100 µL of each dilution were plated on CCA agar for the detection of *E. coli* and *K. pneumoniae* (Chromocult^®^ Coliform Agar, Chromocult, Merck, Feltham, UK); on CHROMagar™ MRSA II (Becton Dickinson and Company, Sparks, MD, USA) for MRSA detection; on Columbia CAN agar with 5% sheep blood (BioMérieux, Marcy l’Etoile, France) for Enterococcus spp. Plates were subsequently incubated for 24 h at 37 °C in aerobiosis. We selected up to four colonies with different morphologies from each sample and subcultivated them in Columbia COS agar with 5% sheep blood (BioMérieux, Marcy l’Etoile, France). Bacterial species were identified with matrix-assisted laser desorption ionization–time of flight mass spectrometry (MALDI-TOF MS, Bruker, Ettlingen, Germany) with the Software IVD MBT Compass v4.2.90. When either *E. coli*, *K. pneumoniae*, *E*. *faecium*, *E.faecalis*, *S. aureus* or *Salmonella* spp. were detected, isolates were subsequently used for DNA extraction, WGS-based typing and characterization of genetic and phenotypic antimicrobial resistances.

### 4.3. Whole Genome Sequencing and Typing

We performed whole-genome sequencing (WGS) on the selected isolates as described previously [69]. High molecular weight (HMW) DNA was extracted using the MagAttract HMW DNA kit (Qiagen, Hilden, Germany). Library preparation was carried out with Nextera XT kit (Illumina, San Diego, CA, USA) and 2 × 250-bp sequencing was carried out on an Illumina MiSeq device. The quality of the FASTQ reads was assessed with FastQC v0.11.9 [70] and the adapters, the last 10 bp of each sequence and sequences below a quality score of 20 were removed or trimmed with Trimmomatic v0.36 [71]. Genome assemblies were generated using SPAdes v3.11.1 [72]. Ridom SeqSphere+ v8.2.0 (Ridom, Münster, Germany) [73,74] was used for WGS-based typing, including species identification, assessment of the genetic relatedness with multilocus sequence typing (MLST) and core genome MLST (cgMLST). For *E. coli*, we used the Warwick cgMLSTv1 scheme hosted at Enterobase, while for *E. faecium* and *E. faecalis* we used the schemes from PubMLST [75,76]. For *K. pneumoniae* we used the stable cgMLST scheme for *K.pneumoniae sensu lato* defined by Seqpshere+. Genome sequences from isolates with unknown alleles and STs were submitted to PubMLST (*Enterococcus sp.*) or EnteroBase (*Enterobacteriaceae*) for the assignment of new alleles and STs by database curators. For each species, MSTs were generated to visualize clusters and the number of allelic differences between the isolates. The default cluster thresholds provided in each of the cgMLST schemes were used. We defined as pseudo-clusters those clusters grouping two or more isolates obtained from the same sample.

ARGs were extracted via the CARD database v4.0 with strict and perfect mode [77]. All ARGs detected were allocated to artificially-created drug classes. Genes encoding for multidrug efflux pumps and other markers whose product conferred resistance to more than one drug class were allocated to the category “multidrug”. In order to plot bar charts with isolate counts the package *ggplot2* v3.3.6 from the R statistical software v.4.1.1 was used [78]. Neighbor-joining trees obtained via Seqsphere displaying the cgMLST of each species were imported into R and visualized with the package *ggtree* v3.4.2 [79]. Afterwards, the *gheatmap* function was used to annotate the trees for the presence or absence of ARGs in each isolate. Virulence genes (VGs) were extracted from the WGS data using the VFDB database v2020 [80]. Plasmids were detected with PlasmidFinder v2.1.1 [81] and the prediction of the possible location (chromosome/plasmid) of acquired ARGs was identified with the Ellipsis pipeline (https://github.com/NorwegianVeterinaryInstitute/Ellipsis). For *K. pneumoniae* isolates, the tool Kaptive v3 was used to identify K capsule types typically associated with hypervirulent (hvKp) clones [82]. For *E. coli* isolates, phylogroups were detected using EzClermont [83] and serogroups with SeroTypeFinder v2.0 [84].

### 4.4. Phenotypic Resistance

Isolates were first screened for resistances by disk diffusion to critically and highly important antimicrobials (see Table S6). For each isolate, a Mueller–Hinton plate prepared in-house was used to inoculate a 0.5 McFarland suspension of the isolate with a disposable swab. Up to six discs with different antimicrobials (Oxoid/ThermoFisher Scientific, Basingstoke, UK) were placed on the plates with a disc dispenser. After 24 h of incubation at 37 °C, the inhibition halos were measured and compared with the EUCAST breakpoint tables for disk diffusion. Possible clavulanate synergy with cefotaxime, ceftazidime or both was assessed in order to look for ESBL producers [85]. We also looked for cefoxitin resistance to identify AmpC producers. Resistant isolates were then tested by E-test (Biomérieux, France) to confirm and quantify (minimum inhibitory concentration or MIC) their phenotypic resistance (Table S7).

An extended antimicrobial susceptibility test (AST) with E-test was also performed for some isolates using additional antimicrobials when acquired ARGs were detected by WGS.

### 4.5. Sequence Data Availability

This whole-genome shotgun project has been deposited in DDBJ/ENA/GenBank under BioProject accession no. PRJNA873263. The version described in this paper is the first version. The raw sequence reads have been deposited in the sequence read archive (SRA). Accession numbers of the *E. coli*, *K. pneumoniae*, *E. faecium* and *E. faecalis* genomes can be consulted in Tables S1–S4.

## Figures and Tables

**Table 1:** Number of isolates gathered by species and compartment in Petzenkirchen, Austria, 2020–2021.

Species	Feed	Field Drainage	Groundwater	Pig Manure	River	Soil	Wastewater	Wildlife	Crops	Total
*E. coli*	1	5	2	1	5	4	12	19	0	49
*E. faecalis*	0	0	1	5	2	3	6	10	0	27
*K. pneumoniae*	0	0	0	0	0	0	7	0	0	7
*E. faecium*	0	0	3	0	0	0	3	0	0	6
Total	1	5	7	5	7	7	28	29	0	89

**Table 2:** Antimicrobial susceptibility patterns of the 26 isolates confirmed as resistant by E-test.

Species	AMR Pattern	Isolates, n (%)	
*E. coli*	AMP/AZT*/CEP/CIP/CTX/ERY*/GEN/MOX*/ STR*/TS*	1	9/49 (18.3%)
	AMP/CIP/MOX*/STR*/TET*/TS*	2	
	AMC/AMP	1	
	STR*/TET*	2	
	AMP	1	
	TET*	2	
*K. pneumoniae*	AMP/FOS*	5	7/7 (100%)
	AMP	2	
*E. faecium*	AMP	1	1/6 (16.7%)
*E. faecalis*	KAN*/STR*/TET*	2	9/24 (33.3%)
	ERY*/TET*	1	
	CLY*/TET*	1	
	TET*	5	

* Antimicrobials used in the second round of E-test for isolates with one or more ARGs possibly linked to phenotypic resistance. AMP = ampicillin, AZT = azitromycin, CEP = cefepime, CIP = ciprofloxacin, CLY = clindamycin, CTX = cefotaxime, ERY = erythromycin, FOS = fosfomycin, GEN = gentamicin, MOX = moxifloxacin, STR= streptomycin, TET = tetracyclin.

## Data Availability

All data are available in the manuscripts or the Supplementary Materials.

## Supplementary Materials

The following supporting information can be downloaded at: https://www.mdpi.com/article/10.3390/ijms231911276/s1**.**
