# Supplementary material for: Characterizing Antimicrobial Resistance in Clinically Relevant Bacteria Isolated at the Human/Animal/Environment Interface Using Whole-Genome Sequencing in Austria
Source: Int J Mol Sci. 2022 Sep 24;23(19):11276. doi: 10.3390/ijms231911276 (PMC9570485; doi:10.3390/ijms231911276)

Figure S1.Number of collected isolates by sampling round, species and compartment

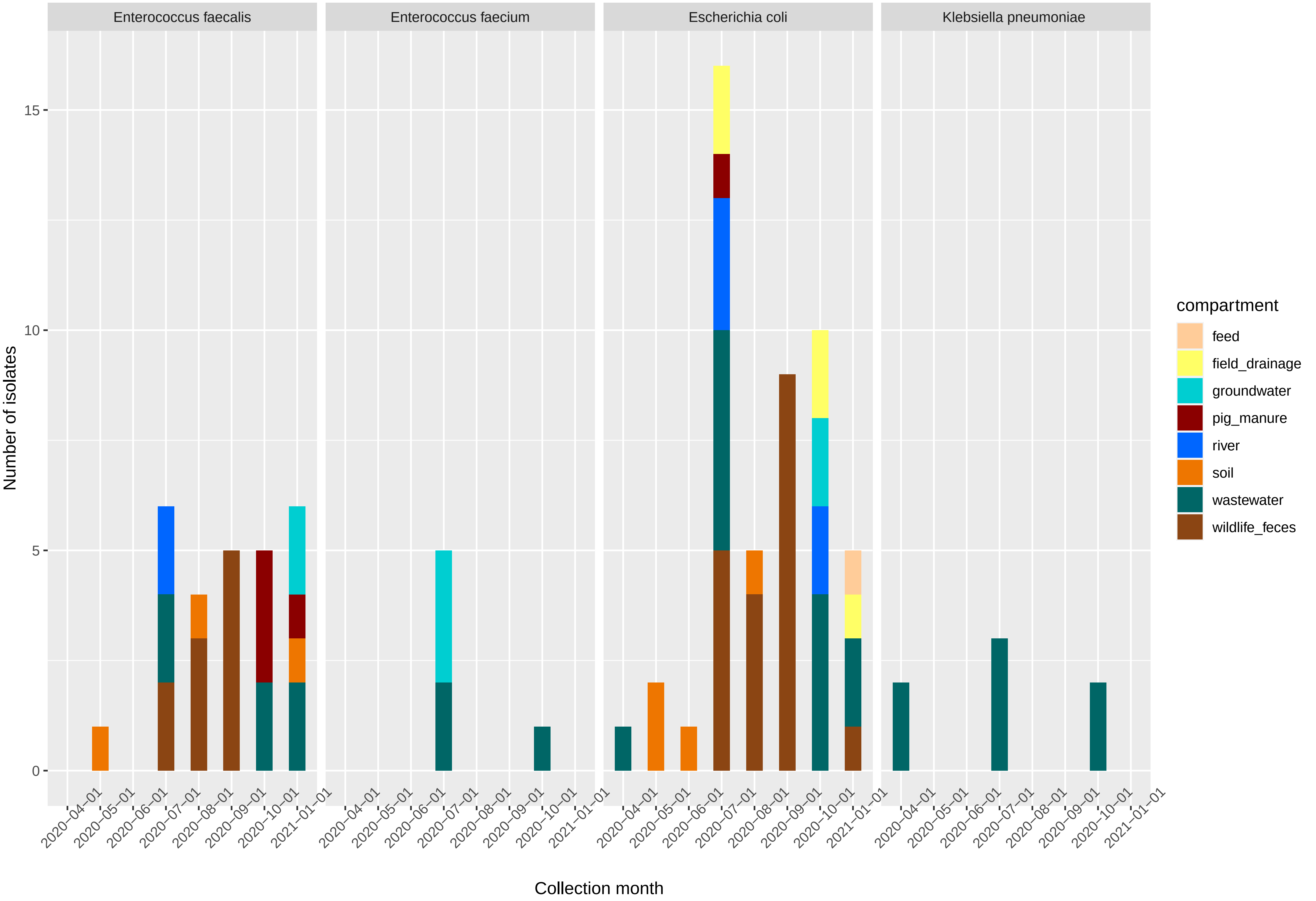

Supplement: Supplementary file 1 [file Figure S1.pdf › Figure S1.pdf]
